# Designing an interoperable patient portal to augment an Advanced Nurse Practitioner service for Children with hydrocephalus

**DOI:** 10.1016/j.ijnsa.2024.100223

**Published:** 2024-07-08

**Authors:** Mary Hughes, Michelle Doyle, Dearbhla Moroney, Orna Fennelly

**Affiliations:** a Trinity School of Nursing and Midwifery, Dublin Ireland; b Neurosurgery Team, Children's Health Ireland at Temple Street, Dublin 1

**Keywords:** Advanced Nurse Practitioner, Children's healthcare, Electronic health record, Patient portal, Interoperability, Hydrocephalus, Digital health, Communication, Chronic illness, Stakeholder consultation

## Abstract

**Background:**

Children's Health Ireland (CHI), who govern and operate acute paediatric services for the greater Dublin area, are also the client for the new children's hospital project which will be Ireland's first fully digital hospital. Design, development and implementation of digital solutions has been prioritised by the National Strategy for Children's Nursing to transform and accelerate nurse-led services.

**Aim:**

The aim of this phase of a larger study was to explore the perspectives and opinions of key stakeholders on the requirements, benefits, and challenges for a bespoke patient portal, with a specific focus on the ANP-led Neurosurgical Service and children and young people with hydrocephalus.

**Methods:**

Interviews and focus groups were held online, and data were recorded and transcribed verbatim. Twenty-three participants across eight sites were interviewed including parents, healthcare professionals, experts and management/administrators. Data were analysed using Braun and Clarke's (2006) framework.

**Results:**

Four key findings and considerations were identified in relation to patient portals in general and their interoperability with Electronic Health Records, as well as a bespoke patient portal for children and young people with hydrocephalus

**Conclusions:**

The availability of a patient portal for children and young people with hydrocephalus would be hugely advantageous to their parents, the ANP led nursing service, and healthcare professionals in both the neurosurgical service at CHI and at regional healthcare organisations as well as for administration, research, and reports. More timely access to health data as well as a consistent log of information and communications between patients and healthcare professionals, would be more efficient and effective than current practices.The augmented ANP-led Neurosurgical Nursing Service at CHI will act as a pilot project from which other nurse-led digital patient services can learn from.

**Study Registration:**

This study was conducted between September 2022 and June 2023. It was registered in Trinity College Dublin, Ireland

**Twitter Abstract:**

A study exploring requirements, benefits, & challenges for an interoperable patient portal in an ANP led Service for children with hydrocephalus

**Summary Points- Contribution of the paper**

**What is Known:** Patient portals are secure online websites or applications that give healthcare service users convenient, 24-h access to their personal health information from anywhere with an Internet connection.

Achieving interoperability of patient portals and electronic health records is challenging due to a myriad of factors which influence the ability of different information systems, devices and applications to access, exchange, integrate and cooperatively use data.

**What this paper adds:** This paper provides guidance for the design and development of a user-friendly and stakeholder informed interoperable patient portal that could be used on a national health service basis.

This paper demonstrates the importance of ensuring interoperability between patient portals and electronic health records as a crucial factor in the successful integration of portal use and adoption with in-person service provision, where equitable and inclusive access and awareness building should also be considered

Alt-text: Unlabelled box

## 1 Background

In Ireland, provision of care to all children and young people with hydrocephalus is primarily provided by the specialist neurosurgical team at Children's Hospital Ireland (CHI) at Temple Street in Dublin. This multidisciplinary team includes an Advanced Nurse Practitioner (ANP), who leads the nursing team of neurological Clinical nurse specialists. Parents and other healthcare professionals involved in care in regional hospitals and the community contact the ANP-led team for advice and information frequently outside of scheduled appointments (Hughes et al., 2023).

Currently, the service is managed using paper-based hospital records for all children, with a variety of information systems for admission and hospital data that are not interoperable in most instances leading to the siloing of information. There is no sharing of medical or nursing records between hospitals other than summary documents such as discharge home forms, leading to multiple health records for children between sites where care is provided and the potential for repeated tests and procedures when this information is not available to clinicians. Children and parents must seek access to their medical chart through Freedom of Information requests (Hughes et al., 2023).

CHI governs and operates acute paediatric services for the greater Dublin area, and leads out on national paediatric services in Ireland (CHI, 2023). It is also the client for the new children's hospital project, which will be Ireland's first fully digital hospital. Following a robust procurement process, Epic Systems Corporation was awarded the tender for the electronic health record (EHR) system, which includes an integrated patient portal. A patient portal is a secure online website or application that gives healthcare service users convenient, 24-h access to their personal health information from anywhere with an Internet connection (HealthIT.Gov, 2017). Using a secure username and password, patients can view health information such as recent hospital visits; discharge summaries; medications; immunisations; allergies; and lab results. Some patient portals also allow secure messaging to healthcare providers; request of prescription refills; scheduling non-urgent appointments; updating of contact information; make payments; downloading and completion of forms; and viewing of educational materials (HealthIT.Gov, 2017). Enabling public access to their health records empowers patients, saves time, improves health outcomes and safety, and improves accuracy, completeness, and readability of records (Antonio et al., 2020; Han et al., 2019; Sipanoun et al., 2022). The integration of this patient portal with an EHR is referred to as interoperability.

Integrating patient portals with EHRs to automatically extract and share the healthcare data with patients and other healthcare organisations has proven challenging internationally (Otte-Trojel et al., 2016). With the development of the new Children's Hospital, Ireland has a unique opportunity to learn and overcome the obstacles experienced by other countries in the development of patient portals. This study identifies key requirements in the design and development of a bespoke interoperable patient portal for the Neurosurgical ANP led-service for children diagnosed with Hydrocephalus. It acts as a template for other advanced nurse/midwife practitioner led services and informs the design of national digital health infrastructure across services and organisations in the health system. Design, development and implementation of digital solutions has been prioritised by the National Strategy for Children's Nursing (CHI, 2021) to transform and accelerate nurse-led services nationally. The ability of the patient portal to transcend geography and augment ANP services to children and families will enable care closer to home, which is one of the pillars of the National Clinical Programme for Paediatrics and Neonatology (2016). This also aligns with Ireland's eHealth Strategy (Department of Health, 2013) and the goals of the Nursing and Midwifery Digital Strategy (ONMSD 2019).

## 2 Aim

The overall aim of this phase of the larger project is to identify and explore the requirements for an interoperable patient portal with a specific focus on the Neurosurgical Advanced Nurse Practitioner Service for children and young people with hydrocephalus.

## 3 Methods

This paper reports on the qualitative findings of a larger study; a scoping review and evidence synthesis which aimed to identify the challenges and requirements for interoperability between patient portals and Electronic Health Records. The Johanna Briggs manual was used as the framework for this scoping review (Aromataris E, 2020); full detail of the literature review from this study is published in another paper (Fennelly et al., 2024). The overall objective of the qualitative enquiry presented here, was to explore the perspectives and opinions of key stakeholders on the requirements, benefits, and challenges for the ANP-led service in incorporating a bespoke patient portal for children and young people with hydrocephalus. Participants included parents of children and young people with hydrocephalus; healthcare professionals involved in the care of these child at both the specialist and regional centres; national experts in digital health development; international experts in digital health development and health service management. A good understanding of the English language was also required to participate in the focus groups and interviews.

Full ethical approval was received from CHI Research Ethics Office, Clinical Research Ethics Committees of three regional Teaching Hospitals, and the Research Ethics Committee [School of Nursing and Midwifery, TCD] All participants offered written informed consent prior to the interview/focus group. Potential healthcare professional participants were invited to participate at each site (n=8) by the site lead and were provided with the appropriate participant information leaflet and consent/assent form (child/parent/Healthcare professional/expert). Participation was entirely voluntary, and they could withdraw at any time. There were different PIL for young people, parents, healthcare professionals, experts and managers/administrators for the purpose of the study. After a minimum of 24 h of consideration, those interested in participating were asked to contact the researchers to ask any further questions regarding the study and if they would still like to participate, arrange a time for their interview. No young people agreed to take part in the study.

### 3.1 Interviews and focus groups

Following a review of the literature and discussions amongst the research team, the interview guides for each stakeholder group were developed. The interview guide differed for each group based on their potential parental/professional contribution to the study. This included the exploration of (i) patient portal information and functionalities; (ii) portal access; (iii) portal interface; and (iv) benefits and challenges of the portal. Virtual interviews and focus groups via Microsoft Teams and Webex, were conducted by two members of the research team; a research physiotherapist [OF] and academic nurse [MH]. Nine middle-aged, native English speaking parents (5 mothers, 4 fathers) were interviewed online, from their own homes at a time that was convenient for them. Online interviews and one focus group were conducted at a time and place in the clinical environment with the healthcare professionals (7 registered nurses with varying amount of post-registration experience, and one senior physician) that was conducive to allowing them to participate. The experts (n=2) and management/administrator participants (n=4) were interviewed online during their working hours, from their desk/workspace at a time convenient for them. Data were collected between December 2022 and March 2023. Recorded data were transcribed by two researchers [OF & DM]. Twenty-three participants were interviewed (Table 1). The duration of interviews and focus groups was a mean of 37 min (Range 22-58 min).

**Table 1:** Stakeholders who participated in the consultation.

**Participant ID**	**Representation**
**P101-109**	Parent of Child/Young Person with Hydrocephalus
**H101**	Nurse at Regional University Hospital
**H102**	Consultant Doctor at Specialist Paediatric Hospital
**H103-108**	Nurses at Specialist Paediatric Hospital
**G01-104**	Management/Administrative Role
**E101**	International EHR Implementation Expert
**E102**	National EHR Implementation Expert

Data were analysed using the Braun and Clarke (2006) thematic analysis process, using NVivo Pro software version 12. The researchers independently familiarised themselves with the transcripts and the initial code set was identified using an iterative approach by one researcher [OF] and reviewed by the second [MH]. Following application of initial code set to a subset of transcripts, amendments were made to reduce any ambiguity of codes and their meanings. During ongoing critical discussions between the researchers, as well as the wider research team, consensus was reached with the two researchers [MH & OF] agreeing on the final themes:

## 4 Results

### 4.1 Balancing benefits & risks of information availability

#### 4.1.1 Useful, transparent information without overload

Participants discussed the type of information and functionalities that should be available in the patient portal including general health and hydrocephalus-specific information (Table 2). While an expert stated that the portal should include *“as much information as possible”* [E101], some healthcare professionals reported that this doesn't need to cover *“every nurses’ note from a whole admission”* [H104] as the healthcare professionals themselves *“don't even want access to their whole health record”* [H102]. They advised that there should be enough information *“just to explain what has happened to their child during their admission, like something that you would give to the child's GP”* [H104]. Some parents also agreed that they didn't think they'd “*want access”* to *“full notes”* as there is a risk of *“overload”* [P103] while another parent had a *“fear of almost seeing the scans*” and recommended a *“health warning”* [P102] if including certain information. A health manager also commented that there was a risk of *“scaring people with all of the information”* [G104]. However, other parents stated that they *“would like to have access to everything”* [P101] and it was acknowledged by several participants that preferences for the level of information will differ between users. Additionally, while there reportedly has to be some standardisation around parameters and release of information, some services will *“have its own particular requirements”* [E102] which was demonstrated in this study as *“all kids present differently”* [P104] and recommendations that their symptoms of being *“shunt sick”* should be provided.

**Table 2:** Information identified for inclusion in the patient portal by participants.

General Information	Hydrocephalus-Specific Information	Functionalities
Discharge Summaries	Type and size of Shunt and valve	Video Consultations
Previous surgeries & outcomes	Type of hydrocephalus	Research
Medications & Prescriptions	Shunt Programme	Prescription renewals
Scans (e.g., Xrays, CTs)	Shunt Series	Appointment scheduling
Laboratory Test Results (e.g., Bloods)	Patient Support Group Links	Secure messaging
Infection status or control issues (e.g., MRSA, VRE)	Educational materials & information (e.g., videos)	Appreciation column for healthcare staff / feedback survey
Underlying diagnoses (and history of diagnoses)	Precautions or contraindications (e.g., MRI compatibility)	Head circumference measurement recording & graph generation
Medical record number (MRN) or other Identifying information	Symptoms of Shunt Malfunction (specific to child)	Reminders
Social History	Type of hydrocephalus	Daily diaries
Hospital admissions	Community/Local Services	
Current Healthcare Providers	Date of shunt insertion & any revisions	
Links & contact details to other healthcare providers (e.g., pharmacies)	Head measurement charts	
Care Plans	Previous shunt revisions	
Appointments	Previous shunt infections or malfunctions	
Vaccines (including schedule)	Parental training	
Referrals & Correspondences	Local training & services	
Contraindications (e.g., MRI compatibility)	Local policy for shunt management	
Biological parents (or legal guardian)	Red flags or checklist	
Previous surgeries and dates	Brand of shunt	
Hospital Information (e.g., maps, guidelines, accommodation)	Travel information & advice (e.g., neurosurgical services abroad)	
Other care needs (e.g., home care package)		

#### 4.1.2 Safeguarding & preventing unnecessary anxiety

In addition to the risk of overloading people with information, the majority of healthcare professionals felt that some information *“should be restricted”* as it *“could create significant anxiety for some parents”* [H102] but should only be done *“for the right reason and protecting the child”* [H101]. Much of this information may only be under discussion, incidental or not relevant but cause parents to *become “heightened with worry because this information is in black and white in front of them”* [H103] and “at *that stage it's like 2 and 2 can add up to 10”* [G101]. Other cases where it was discussed that it may not be appropriate to automatically share information was in cases where the patient and/or healthcare professionals needed to be safeguarded. For example, *“there could be allegations of child abuse”* [H105] and the healthcare professional making the allegation might also need to be protected. To overcome these challenges with data release, standardised parameters were recommended for the release of test results such as releasing only once *“discussed with the parents”* [G104] and/or released after a few weeks.

#### 4.1.3 Timely & trusted access to information

A *“huge advantage”* [H101] of a patient portal according to participants, was the *“timely access”* [G101] which would save time for everyone by providing accurate information and benefit the patient directly. This would allow the patients to share the information directly with the ANP or other healthcare professionals in both the community and the hospitals *“Our pharmacist when they come and do reconciliation and the children's medications. And the mother is going but I thought there was this dose, and it wasn't this dose…I mean there's your problem solved straight away and there's a query and a concern sorted”* [H101]. Additionally, access to this information from home would reportedly *“empower parents”* [H104], *“makes them feel valued”* [E101], *“be life saving for some child”* [P106] and act as *“reminders”* [P101] for appointments and medical history. At present, many parents spoke about the *“Bible”* [P103] of paperwork that they have on their child as well as the paper shunt card with the child's information which was often reportedly damaged or lost. This portal would allow them to concentrate more on caring for their child and make them more comfortable to bring their child on holidays and to a hospital “closer to us but we go to *specialist hospital* because it's just that's where his records are” [P102]. It would allow the child to safely be away from home as *“at least if he had something on his phone, everything is there that he can you know, tell people if he needs to tell people or if they're even in school”* [P103]. Finally, the information shared on the portal would be trusted by parents and allow them to learn about hydrocephalus and shunt management from trusted sources rather than *“Google”* [P106] which would *“frighten”* [P107] people.

### 4.2 Usable & accessible portal interface

#### 4.2.1 Accessible & inclusive portal

The accessibility of the patient portal for everyone of all abilities was highlighted by parents, healthcare professionals, experts and health service managers. Access to the portal via a mobile phone was the preference of most parents *“as you'll always have your phone with you”* [P101] with both IOS and Android compatibility recommended. However, it should be noted that there could be an *“equitability challenge”* [G101] as not everyone may have internet access or a phone with the capability to download the portal app which result in *“missing a lot of children whose parents can't afford the decent phone with the portal”* [G104]. Other challenges to accessing the portal and information included health literacy levels, intellectual disabilities, language barriers, visual deficits and perception issues. According to parents of children with hydrocephalus, there will be different needs with one parent discussing the challenges for their child being nonvisual. It has been recommended that *“if you are a person who has maybe perception issues or language issues, maybe a graphic is the best way to go”* [H101] and *“videos that are kind of easy for people with visual difficulties”* [P102]. However, preferences will differ between users with some preferring *“written word…find a video harder to watch”* [P101] and others stating *“infographics and visual is probably best digested by me”* [P102]. Other recommendations to promote inclusivity included “*different language options”* [H101], removing medical *“jargon”* [G104], linking a *“national resource to explain what the test results are”* and ensuring the information is *“aimed at the person”* but this is *“quite a cultural change”* [E101].

#### 4.2.2 Simple & easy to use interface

To promote adoption and valuable use of the patient portal, it must be *“as simple as possible”* [H104] and *“user-friendly”* [P103] according to participants with acknowledgement that some people will be more *“tech-savvy”* [P102] than others. An expert [E101] with experience in the delivery of a patient portal reported that *“after two years we'd only got 2000 patients signed up on…so we changed quite a lot, but some of what we changed were the activation process because we realized we'd made it harder than general access to health records”*. A patient [P103] also highlighted *“that would really only work if it was really easy to access it really quickly, like if I had to go through log on and then I have to go through five or six different pages to get to where I need to get. I don't think that's gonna be useful then, because it gets too complex. Ah here just give me a sheet of paper and I'll write it down”.* Recommendations for a simple easy-to-use portal included only having a *“few tabs”* [P102], a *“very definite menu”* which is *“less fussy”* [H101], *“steps and signposts”* and *“one click and you're in”* [G104], and *“quick”* [P103] to access.

### 4.3 Communications, integration & data generation

#### 4.3.1 Communication & support from experts

For patients with shunts and their families, communication with and the support and care provided by the ANP- led team was reportedly critical for them especially after initial diagnosis. *“They would be our go to people, you know, if we felt like we needed something urgently, we would definitely contact the neuro team”* [P106]. This involved *“phone calls every couple of days”* [P101] or emails, to *“reassure”* [P102], “discuss *the educational material* further” [H103], or *“give us heads up that they're on the way in”* [H104]. The interviewed parents trusted the ANP-led team and reported that they *“felt so safe the whole time”* [P101] and had *“built a big bond”* [P107] and *“a good relationship”* [P108] with them. This need for support also stemmed from *“a lack of confidence in the ability of their local ED to manage it”* [H105] with parents feeling that their local hospital *“doesn't have the knowledge about shunts”* [P103]. However, the ANP-led nursing team or neurosurgical team recommend them to attend their local hospital who can then also ring the team at CHI for further information. While the parents felt communication via the portal would be beneficial, many parents and healthcare professionals did not think this would completely replace the phone calls as *“there's nothing like the human voice at the other end of the phone and just trashing it out”* [P103] and *“a lot of parents do like the reassurance that you can give verbally to them”* [H106]. However, as time goes on, the parents reportedly become the experts in their child's management, and they could see where a secure message via the portal would be beneficial to *“just flag it up”* [P103] and *“avoid ringing them to bother them”* [P106]. The other benefits of the communication via the portal would be if “anyone in his team could have access to *message* if *Advanced Nurse Practitioner* is on holidays” [P104], a *“log”* [P105], avoid anyone *“falling through the loop”* [H103] and *“getting answers to your questions from the comfort of your own home and request your repeat prescriptions”* [E102].

#### 4.3.2 Responsibility, workload & patient safety

Concerns were raised in relation to the secure messaging service creating additional workload requirements and being a risk to patient safety. Healthcare professionals would reportedly like to see the message box provide an automatic reply to parents saying *“if you're not getting a response and you're worried about your child, get them to your nearest ED”* [H105]. Similarly, other participants highlighted that that if there was no response *“they're gonna be like what's the point”* [E101] and there should be a way of knowing the message was *“accepted or you know that somebody has been able to follow it up”* [G104] or *“accessed the information”* [P101]. Similarly, safety risks were also discussed in relation to the use of Chatbots within the secure messaging. There were also *“some ethics and legal issues to work through in terms of if they send it is who's obliged to look at it”* [E101] and *“how we manage the data and share it and store it going forward and then we have to delete it…how long does that stay on the portal and who's responsible to delete it”* [H101]. To avoid *“alert or communication fatigue”,* it was recommended that *“some sort of parameters put in around that”* [E102]. Additionally, prior experiences have reportedly demonstrated that activity was low and *“it stopped phone calls”* and as the messages were recorded in the Electronic Health Record, “*they didn't need to transcribe anything”* [E101].

#### 4.3.3 Valuable data generation & capture

It was acknowledged that the patient portal could generate a lot of valuable data in the form of integrated devices, daily diaries, patient-reported outcome measures (PROMs), pre- and post-clinic surveys, vital signs, videos, photographs, research data, reports and analysis. *“Anything's possible generally in a technology perspective and I think in the longer term, a lot of wearables will, people will be able to send in results”* [E101]. However, this should follow a *“standardised approach”* [E102] with people capturing *“information in a more succinct way that can be utilised”* [G101]. Additionally, the data collected via the portal should be valuable and usable and *“not data for data sake either*” [G101] or *“laborious”* [G102] to collect. Additionally, *“rules and regulations”* [E102] will be required for ethical research to be conducted using these data. Capturing data using the patient portal could reportedly benefit the healthcare professionals in *“knowing all of that in advance of when you're going to see them and know what what's wrong with them now”* [E101]. While for parents, it would be a lot easier if they could *“take a photograph of a wound and just upload it through the app”* [P105] with *“that information going straight into the team”* [P101]. Similarly, capture of administrative, audit and research data would allow improvement of care, information provision and evaluation of services, for example *“what are those frequently asked…maybe we're not giving enough information on our website”* [G104] and with a central log of patient communications you can *“see if there's things that are reoccurring, or what needs to be addressed”* [H103].

### 4.4 Data protection, user access & interoperability

#### 4.4.1 Data sharing & interoperability

Interviewed parents were in agreement that healthcare professionals, *“where it's medically necessary”* [P105], *“should have access straight away, so they know what they're dealing with”* [P107]. This would also support parents in educating healthcare professionals, who were *“not very aware”* of their health condition, to “understand *child* you know as much they can from his records” [*P102].* This data sharing with another healthcare provider would also be critical during the transition to adult services according to parents and *“that interoperability piece” will be important to “transition seamlessly across so they don't lose”* [G103]. Health service managers acknowledged challenges with other healthcare organisations accessing the patient portal due to *“governance, accountability for that information sharing…going from one organisation to another”* [G101] and different types of organisations such as “*HSE and voluntary section 38”* [G104] complicating the data sharing process*.* It was reported the digital infrastructure to support this data sharing was lacking and negotiation and collaboration with vendors and the different stakeholders would be required. Additionally, *“a proper legislative and GDPR compliant agreement and then ASLA”, “compliance with the eHealth Strategy”* [E102] as well as single patient identifiers were identified as required to support this. “At the moment, *child* has 3 hospital numbers…And again, that's just ridiculous, like frankly it's just ridiculous, he doesn't need a 4th or a 5th one, he just needs 1” [P105].

#### 4.4.2 Consent, child & proxy access

It was recommended that the clinical team was not put in charge of choosing who was provided with access as *“it wasn't high on their list of priorities to deal with when they've got that person in front of them”* [E101]. In this case of a paediatric hospital, proxy access was critical, and this access needed to align with a *“legal framework”* and *“consent law”* and involve “*open and honest conversations with those that are becoming young adults”* [E101]. However, there will be cases where *“lack of capacity to actually to engage*” [E102] needs to be considered with some being *“fully functional, cognitively verbal kids*” [P103] and others not. Other considerations included *“if your parents were separated”* [H101], with an individual *“login per parent*” [P102] suggested. Once the child or young person was *“responsible for their own health”* [H101], they will need *“to understand why she has the shunt and how to look after herself with it”* [P102]. In conjunction with consent law, permitting access to the child should reportedly be *“down to each individual family”* [P103] to determine *“the right age”* [P101] as “*you may have a very mature 12-year-old or 10-year-old, but you have a very immature 13 or 14 year old”* [G104]. This should also be a *“gradual transition…start when they're 15 and give them basic information”* [H101] which could be assisted by *“resources they could share with their child”* [H104], *“tailored material for them and their age”* [P102] and *“restricted access for kids”* [H106]. However, concerns were raised where parents no longer have portal access as they may not know *“when the follow up appointments that the children going, are they taking the medication, have they filled their prescriptions”* [H101]. It was also recommended that the child accessing the portal should *be “flagged with somebody”* [G104].

#### 4.4.3 Security of data & devices

The importance of cybersecurity for the patient portal was discussed by most participants and many referenced the recent cyberattack on the public health service in Ireland. While there were concerns raised by both parents and healthcare professionals that *“everyone's worried about the hack….and whether they can trust us with their information”* [H101], the parents interviewed for this study were largely supportive of the portal. *“Setting up this portal is far more beneficial than the thoughts of not setting it up based on a possible data breach. I wouldn't be overly concerned, I kind of put my faith in the powers that be the security Wiz kids”* [P101]. Some parents also discussed the security risks which already exist with paper records. *“They actually lost some of the paperwork….if things were digital that wouldn't happen”* [P102]. However, parents and healthcare professionals alike discussed nervousness of everything being online if there was another cyberattack and the need for a “*security blanket*” [H106] if this happened to support “*access without being online”* [P107]. Healthcare professionals and other healthcare providers also discussed the importance of “*reassuring patients and parents that the connection is good and the connection is safe”* [G104]. Additionally, the security of the device used to access the portal was also discussed by a healthcare professional. *“Who's going to have access to their devices and make sure that they have an access and ability to lock it”* [H101]. Portal passwords and being able to reset passwords were also important security features mentioned by parents. One health service manager did however raise the risk of *“cyberwalls”* [G101] between systems inhibiting data sharing resulting in data loss too.

## 5 Discussion

These findings suggest that the availability of a bespoke patient portal for children and young people with hydrocephalus would be hugely advantageous to their parents, the ANP-led nursing service, and healthcare professionals in both the neurosurgical service at CHI and at regional healthcare organisations as well as for administration, research, and reports. More timely access to health data as well as a consistent log of information and communications between patients and healthcare professionals, would be more efficient and effective than current practices.

Engagement with key stakeholders has resulted in a robust set of recommendations for the team deigning the electronic health record and patient portal. This will have benefits for the wider health service as it moves towards a digital health enabled service for the whole population. It will also inform the ongoing expansion of Advanced Practice services led by nurses and midwives across the country. Managing patient caseloads and integrating real time data in patient caseload management will enable quality improvement data specific to the service. This is not currently available as activity is captured under the medical directorate lead/consultant in the current system. Coding of activity by Advanced Practitioners will be feasible if the system is designed accordingly. Based on the findings from this phase of the larger study, the following key findings and considerations were identified in relation to patient portals in general and their interoperability with Electronic Health Records, as well as a bespoke patient portal for children and young people with hydrocephalus.

### 5.1 Personal health information availability

Balancing the type and level of information available requires engagement and collaboration with the key stakeholders including patients and families, the ANP-led nursing service and healthcare professionals, with some standardisation across healthcare organisations is recommended. However, there will be specific requirements for this population who need to have access to, and the ability to share their shunt-specific information. While transparent and open release of information was identified as important and is required under the GDPR, release of entire health records within the patient portal may not always be appropriate or beneficial. To safeguard and protect users from overload and unnecessary anxiety (Sipanoun et al., 2022), healthcare summaries may be released and there may be restrictions in place on release of test results until reviewed by a clinician (Essén et al., 2017; Pearce and Bainbridge, 2014; Wald et al., 2004) or on information to safeguard patients and healthcare professionals (Scandurra et al., 2017; van Kuppenveld et al., 2020; Zanaboni et al., 2020).

### 5.2 Technical & workflow integration

While the healthcare professionals within the ANP-led Neurosurgical service and the parents of children with hydrocephalus reported extensive communication via phone calls and emails especially when the child was first diagnosed, use of secure messaging and other data coming in via the portal will need to be integrated into the healthcare provider workflows (Sipanoun et al., 2022) with parameters set in terms of the monitoring of this message box and data to ensure patient safety (Anoshiravani et al., 2011; Hess et al., 2007). Additionally, existing software such as appointment scheduling would need to be integrated with the patient portal to allow immediate updating and support this functionality within the portal (Otte-Trojel et al., 2015; Wald et al., 2004). The extensive potential to gather data from patients and portal interactions also requires consideration of the value of the data being collected, responsibility over analysis and use, and the storage and management of the data.

### 5.3 Authentication, access & interface

To share the data via a portal, clear consent procedures are required with consideration of age, capacity and proxies. Additionally, access to the patient portal or a similar shared care record by the ANP-led nursing service or other healthcare professionals involved in provision of care requires consideration of supporting control over this access by the data owner. To ensure the correct person is provided access to the correct record, identifiers and verification procedures have been employed internationally, but unique identifiers used across all healthcare organisations were used to avoid use of several patient portals if attending a number of different healthcare providers (Barbarito et al., 2015; Cunningham et al., 2013; Lämsä, et al., 2017; Pearce and Bainbridge, 2014; M. Sääskilahti, et al., 2021; Santos et al., 2021; Scandurra et al., 2016; Scandurra et al., 2017).

To promote adoption of the portal, simple user-friendly access and interface are recommended as well as consideration of equitable access (Antonio et al., 2019). This study as well as other literature recommends access via mobile phones (Bush et al., 2017; Bush et al., 2016), translation to other languages (Bush et al., 2017), plain language (Geerts et al., 2019; Zhang et al., 2020) and simple screens and clear navigation (Bush et al., 2016; Smith et al., 2017). This study also strongly supports consideration of those with intellectual disabilities and visual impairments in the development with consideration of visual and non-visual aids and other supports. Application of plain language will require a culture change among the healthcare professionals entering data but linked resources explaining results have also been used (Lau et al., 2014; Pearce and Bainbridge, 2014; Ro et al., 2015; Wald et al., 2004).

Awareness of the portal among the service users should also be promoted. Overall, consent policies, unique identifiers, a user-centred and friendly portal design, and awareness building campaign are recommended for the implementation of this portal.

### 5.4 Data sharing, security & data protection

While the patient portal at CHI will not support direct access by other healthcare organisations in the first instance due to a need for digital infrastructure and vendor agreements, a number of key considerations for data sharing, security and data protection were identified. Participants in this study reported concerns with data security when sharing data but overall, the benefits outweighed the risks especially for these young people with hydrocephalus who will transition to adult services and will want to bring their health records with them. Additionally, direct access to the health record by healthcare professionals at regional centres was a preferred mechanism within this study but this would require the digital infrastructure to be in place as well as consent procedures. Many studies in the literature review allowed users to see and control who had access to their health record (Pearce and Bainbridge, 2014). Security measures will also be critical which included password protection, encryption and testing.

## 6 Limitations

While this project focused on children and young people with hydrocephalus and the neurosurgical ANP-led nursing service at CHI, it has implications for patient portals across Ireland and internationally. Unfortunately, viewpoints from only one regional hospital were obtained during the stakeholder consultations and further consideration should be given to the primary care and community voice. In addition to this, no young people came forward to participate in the study.

## 7 Conclusions

Overall, this consultation identified key requirements for the ANP-led nursing service with a bespoke patient portal for children and young people with hydrocephalus as well as the patient portal for all CHI service users and future patient portals in Ireland and elsewhere. While acknowledging the potential benefits, there are many considerations to ensure the investment in and use of the patient portal is optimised. Parameters and criteria for consent, access, information and functionality availability as well as security and data protection are recommended and could be applied at a national level. To promote the further expansion and innovation in ANP-led services integrating portal use and adoption with in-person provision, equitable and inclusive access and awareness building should also be considered. To avoid users requiring access to several different patient portals in the future, the building blocks for a national patient portal should be considered at this time which may include incentives for data sharing, data standards, unique identifiers and collaboration and engagement with the Health Service Executive and all healthcare providers.

## CRediT authorship contribution statement

**Mary Hughes:** Writing – original draft, Project administration, Methodology, Investigation, Funding acquisition, Formal analysis, Data curation, Conceptualization. **Michelle Doyle:** Writing – review & editing, Investigation, Funding acquisition. **Dearbhla Moroney:** Writing – review & editing, Project administration, Methodology, Investigation, Formal analysis, Data curation. **Orna Fennelly:** Writing – review & editing, Methodology, Investigation, Formal analysis, Data curation.

## Declaration of competing interest

No conflict of interest to declare.

## Appendix Supplementary materials

Image, application 1

Image, application 2
