# Supplementary material for: Designing an interoperable patient portal to augment an Advanced Nurse Practitioner service for Children with hydrocephalus
Source: Int J Nurs Stud Adv. 2024 Jul 8;7:100223. doi: 10.1016/j.ijnsa.2024.100223 (PMC11315161; doi:10.1016/j.ijnsa.2024.100223)
Supplement: Supplementary file 1 [file mmc1.docx]

| **Participant ID** | **Representation** |
| --- | --- |
| **P101-109** | Parent of Child/Young Person with Hydrocephalus |
| **H101** | Nurse at Regional University Hospital |
| **H102** | Consultant Doctor at Specialist Paediatric Hospital |
| **H103-108** | Nurses at Specialist Paediatric Hospital |
| **G01-104** | Management/Administrative Role |
| **E101** | International EHR Implementation Expert |
| **E102** | National EHR Implementation Expert |

*Table 1: Stakeholders who participated in the consultation*

Table 2 Information Identified for Inclusion in the Patient Portal by Participants

| General Information | Hydrocephalus-Specific Information | Functionalities |
| --- | --- | --- |
| Discharge Summaries | Type and size of Shunt and valve | Video Consultations |
| Previous surgeries & outcomes | Type of hydrocephalus | Research |
| Medications & Prescriptions | Shunt Programme | Prescription renewals |
| Scans (e.g., Xrays, CTs) | Shunt Series | Appointment scheduling |
| Laboratory Test Results (e.g., Bloods) | Patient Support Group Links | Secure messaging |
| Infection status or control issues (e.g., MRSA, VRE) | Educational materials & information (e.g., videos) | Appreciation column for healthcare staff / feedback survey |
| Underlying diagnoses (and history of diagnoses) | Precautions or contraindications (e.g., MRI compatibility) | Head circumference measurement recording & graph generation |
| Medical record number (MRN) or other Identifying information | Symptoms of Shunt Malfunction (specific to child) | Reminders |
| Social History | Type of hydrocephalus | Daily diaries |
| Hospital admissions | Community/Local Services |  |
| Current Healthcare Providers | Date of shunt insertion & any revisions |  |
| Links & contact details to other healthcare providers (e.g., pharmacies) | Head measurement charts |  |
| Care Plans | Previous shunt revisions |  |
| Appointments | Previous shunt infections or malfunctions |  |
| Vaccines (including schedule) | Parental training |  |
| Referrals & Correspondences | Local training & services |  |
| Contraindications (e.g., MRI compatibility) | Local policy for shunt management |  |
| Biological parents (or legal guardian) | Red flags or checklist |  |
| Previous surgeries and dates | Brand of shunt |  |
| Hospital Information (e.g., maps, guidelines, accommodation) | Travel information & advice (e.g., neurosurgical services abroad) |  |
| Other care needs (e.g., home care package) |  |  |
