# Supplementary material for: Designing an interoperable patient portal to augment an Advanced Nurse Practitioner service for Children with hydrocephalus
Source: Int J Nurs Stud Adv. 2024 Jul 8;7:100223. doi: 10.1016/j.ijnsa.2024.100223 (PMC11315161; doi:10.1016/j.ijnsa.2024.100223)
Supplement: Supplementary file 2 [file mmc2.docx]

**Ethics Statement**

Full ethical approval was received from CHI Research Ethics Office, Clinical Research Ethics Committee of three regional Teaching Hospitals, and the School of Nursing and Midwifery Research Ethics Committee, Trinity College Dublin. All participants offered written informed consent prior to the interview/focus group. Potential participants were invited to participate at each site (n=8) by the site lead and were provided with the appropriate participant information leaflet and consent/assent form (child/parent/Healthcare professional/expert). After a minimum of 24 hours of consideration, those interested in participating were asked to contact the researchers to ask any further questions regarding the study and if they would still like to participate, arrange a time for their interview. Participation was entirely voluntary, and they could withdraw at any time.
